# Modeling and Optimization of Triticale Wort Production Using an Artificial Neural Network and a Genetic Algorithm

**DOI:** 10.3390/foods13020343

**Published:** 2024-01-22

**Authors:** Milana Pribić, Ilija Kamenko, Saša Despotović, Milan Mirosavljević, Jelena Pejin

**Affiliations:** 1 Department of Biotechnology, Faculty of Technology Novi Sad, University of Novi Sad, Bulevar cara Lazara 1, 21000 Novi Sad, Serbia; milana.pribic@uns.ac.rs (M.P.); jpejin@uns.ac.rs (J.P.); 2 The Institute for Artificial Intelligence Research and Development of Serbia, Fruškogorska 1, 21000 Novi Sad, Serbia; 3 Department of Food Technology and Biochemistry, Faculty of Agriculture, University of Belgrade, Nemanjina 6, 11080 Belgrade, Serbia; sdespot@agrif.bg.ac.rs; 4 Institute of Field and Vegetable Crops, Maksima Gorkog 30, 21000 Novi Sad, Serbia; milan.mirosavljevic@nsseme.com

**Keywords:** triticale, adjuncts, mashing, artificial neural network, genetic algorithm

## Abstract

Triticale grain, a wheat–rye hybrid, has been reported to comply very well with the requirements for modern brewing adjuncts. In this study, two triticale varieties, in both unmalted and malted forms, were investigated at various ratios in the grist, applying different mashing regimes and concentrations of the commercial enzyme Shearzyme^®^ 500 L with the aim of evaluating their impact on wort production. In order to capture the complex relationships between the input (triticale ratio, enzyme ratio, mashing regime, and triticale variety) and output variables (wort extract content, wort viscosity, and free amino nitrogen (FAN) content in wort), the study aimed to implement the use of artificial neural networks (ANNs) to model the mashing process. Also, a genetic algorithm (GA) was integrated to minimize a specified multi-objective function, optimizing the mashing process represented by the ANN model. Among the solutions on the Pareto front, one notable set of solutions was found with objective function values of 0.0949, 0.0131, and 1.6812 for the three conflicting objectives, respectively. These values represent a trade-off that optimally balances the different aspects of the optimization problem. The optimized input variables had values of 23%, 9%, 1, and 3 for the respective input variables of triticale ratio, enzyme ratio, mashing regime, and triticale variety. The results derived from the ANN model, applying the GA-optimized input values, were 8.65% *w*/*w* for wort extract content, 1.52 mPa·s for wort viscosity, and 148.32 mg/L for FAN content in wort. Comparatively, the results conducted from the real laboratory mashing were 8.63% *w*/*w* for wort extract content, 1.51 mPa·s for wort viscosity, and 148.88 mg/L for FAN content in wort applying same input values. The presented data from the optimization process using the GA and the subsequent experimental verification on the real mashing process have demonstrated the practical applicability of the proposed approach which confirms the potential to enhance the quality and efficiency of triticale wort production.

## 1. Introduction

Brewing is one of the oldest food processes that began in the Middle East 10,000 years ago and currently is one of the leading food industries in the world. Over the last thirty years, there has been a remarkable increase in the demand for beers produced on a small scale and via a slower fermentation process, namely craft beers. One of the main characteristics of craft beers is the utilization of non-standard cereals [1,2,3].

Beer is typically brewed using raw ingredients including malt, yeast, hops, and water, which are transformed during consecutive and complex technological processes [3]. Traditionally, barley, in its malting form, is a common choice. The three main steps that can ensure biochemical changes occur within the grain during malting are steeping, germination, and kilning. The production of enzymes in the germinating cereal grain, which causes changes in the chemical constituents of barley in preparation for mashing, can be stated as the main aim of malting [4]. However, the enzymes developed during malting do have some limitations, as they can only work in certain environments, and their activity might be too low to properly degrade certain substances [5,6]. In comparison with endogenous enzymes, commercial enzymes are more active and stable at preferred mashing conditions. The addition of commercial enzymes can make the brewing process far more consistent and can create the opportunity to use cheaper or poorer quality raw materials [5]. With the above in mind, it can be assumed that today’s brewing cannot continue without the utilization of commercial enzymes [7].

As the malting process is not economically efficient, brewers often seek various strategies for cost reduction. Accordingly, the use of barley malt substitutes, as a source of extract, can be part of the solution. These materials, which bring additional sources of carbohydrates and protein into the wort, are called adjuncts [1,8,9].

The main disadvantage in terms of adjunct processability is their lower levels of cytolytic, proteolytic, and amylolytic enzymes compared to barley malt. However, there are a few examples of deviations from this rule, as some adjuncts, among which is triticale, may provide further enzymatic activity [8,10]. Even in its unmalted form, triticale can produce high levels of α-amylase. Besides that, having a low starch gelatinization temperature (59–65 °C) brings an advantage in achieving efficient starch degradation, similar to barley malt [11,12].

Of all beer production stages, mashing is a crucial step, since it determines the composition of the wort, which has a major influence on yeast fermentation and final beer quality [13,14]. Grist ratios, mashing temperatures, as well as the time required for enzymes present in grains to convert specific substances, have to be adjusted to accomplish the optimal mashing process [14]. Since modern brewing practice requires modified mashing in terms of applied temperatures, owing to the increased amount of adjuncts in the grist, the standard Congress mashing method could be questioned [15].

In the past, food-related studies were conducted using more conventional approaches. However, nowadays, the development and adoption of new technologies are dramatically accelerated. This rapid technological change affects almost all areas of life, including the food industry [16].

The implementation of novel modeling and optimization methods in brewing holds the promise for enhancing economic efficiency without compromising the distinct sensory characteristics of the beer. For this reason, an artificial neural network (ANN) can be used. In recent years, the application of ANNs has become a useful tool to increase accuracy and time, and reduce costs in analytical methods. Therefore, ANN modeling has the potential to solve problems by bypassing traditional approaches [17,18].

The brewing process, as a bioprocess, can be illustrated by empiric mathematical models, due to variable interactions and complex biochemistry reactions, which require efficient the modeling methodologies. ANNs are distinguished by their nonlinear aspect; therefore, they can detect complex nonlinear correlations between dependent quality parameters and independent process parameters. The advantage of employing neural networks lies in the fact that without having exhaustive knowledge about a specific process, the network can learn from previous experiences to predict the system’s behavior when some variables are modified. To make the selection of ANN topology more accurate, which depends on various features such as a number of hidden layers, number of neurons of hidden layers, and connections among neurons, using evolutionary algorithms can be a valuable asset. As such, GAs have been applied in studies of bioprocess optimization from experimental data using ANNs, to optimize their topologies and to deal with the optimization of multiple objective functions [17,18]. The concept of many-objective optimization has emerged as a powerful approach to efficiently explore the trade-offs between multiple objectives and identify a set of solutions that represent the best compromise solutions [19]. GAs mimic the biology of chromosome generation with operators such as mutation, crossover, and the selection criteria of the random population of individuals. By applying an objective function, each individual is evaluated based on a fitness criterion [20].

ANNs and GAs showed remarkable ability in modeling and optimizing various processes. Specifically, within the brewing industry, these artificial intelligence techniques were effectively employed to enhance fermentation processes [21]. A dynamic neural network was developed, comprising adapted neurons to account for process dynamics. This model accurately captured the intricate, non-linear nature of beer fermentation, enabling predictions of future process behaviors. Also, an ANN was used in beer classification to build a classification model based on the relevant features of beer [22]. As one of the most relevant components of beer taste, the control of acetic acid content is very important to maintain consistent beer quality. For that reason, an ANN and a support vector machine were applied to predict acetic acid content at the end of commercial-scale beer fermentation [23]. ANNs and GAs were not only applied in the brewing process, but also to optimize the further utilization of brewing bio-waste materials such as brewers’ spent grain [20], where the biosynthesis of poly-β hydroxybutyrate from brewers’ spent grain was modeled and optimized using an ANN–GA strategy. Besides that, better control of a brewery’s waste water was achieved by developing a robust mathematical tool for performance prediction using an ANN [24].

Although ANNs and GAs have commonly been utilized in studying the final fermentation process in brewing, there remains a gap in research regarding their application in modeling and optimizing the production of wort. Therefore, this study aimed to develop a new approach to the mashing process, applying an ANN model that used the data obtained from a laboratory wort production of two triticale varieties—NS Paun and Odisej—in unmalted and malted forms. The conventional Congress mash protocol and a modified temperature variation regime, with and without the application of a commercial enzyme, were explored. Moreover, a multi-objective optimization approach based on a GA was developed to optimize the conditions needed to obtain the best wort quality.

## 2. Materials and Methods

### 2.1. Materials

Two winter triticale varieties—NS Paun and Odisej, both accepted in Serbia and the European Union—were examined in unmalted and malted forms. They were grown in experimental fields in Rimski Šančevi, Serbia. As a control sample, commercial barley malt type Pilsner, produced by malthouse Maltinex Soufflet Group, Bačka Palanka, Serbia, was used. Commercial enzyme Shearzyme^®^ 500 L as xylanase (endo-1,4-; produced by *Aspergillus oryzae*) was kindly provided by Novozymes A/S (Bagsvaerd, Denmark). For free amino nitrogen (FAN) content determination, the following chemicals were purchased from Sigma-Aldrich: disodium hydrogen phosphate dodecahydrate, potassium dihydrogen phosphate, ninhydrin, fructose, potassium iodate, ethanol 96%, fructose, and glycine.

### 2.2. Methods

**Figure 1:**
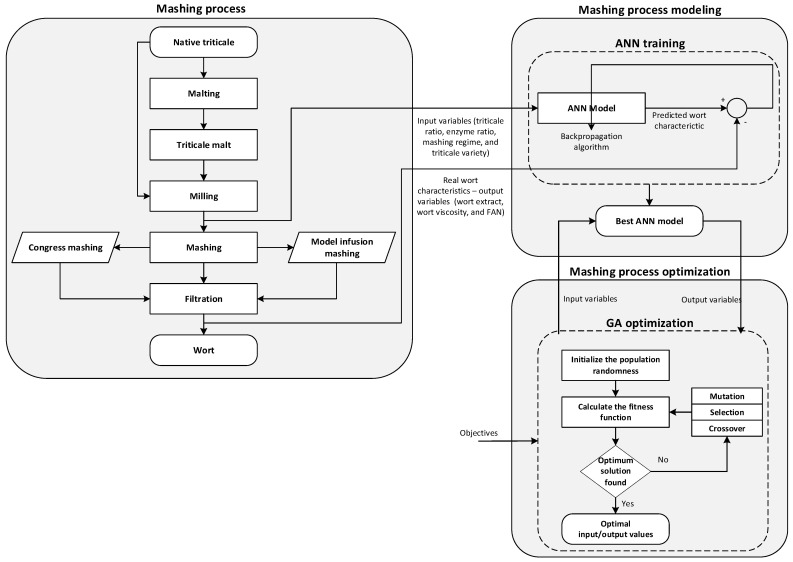
Flowchart of mashing, modeling, and optimization of triticale wort production.

The flowchart in Figure 1 summarizes the overall process of mashing, modeling, and optimization of triticale wort production.

#### 2.2.1. Mashing Process

The mashing process consists of several successive steps, as can be seen in Figure 1. In this study, triticale was utilized in two forms—unmalted and malted cereal. In order to produce malt, a micro-malting pilot plant consisting of a steeping, germination, and kilning unit (Seeger, Germany, at the Faculty of Technology, Novi Sad, Serbia), with the following malting program: 3 days of steeping with a wet and dry cycle at 15 °C (1st day—6 h wet, 18 h dry; 2nd day—4 h wet, 20 h dry; and 3rd day—2 h wet, 22 h dry) was used. The germination phase lasted 4 days at 16 °C. After that, green malts were dried by applying the following kilning temperatures: 55 °C for 15 h, 72 °C for 5 h, 82 °C for 3 h. The rootlets generated during germination were eliminated from the malts by deculming.

To quantify the main wort quality indicators, cereal samples were finely milled in a laboratory disc mill, DLFU (Bühler GmbH, Braunschweig, Germany), at 0.2 mm. Mashes were prepared in mechanically stirred metal beakers in a mash bath (Lochner Labor Technik GmbH, Berching, Germany) using 50 g of grist in total mixed with 200 mL distilled water. After the mashing process, to obtain extracted liquid wort, mashes were filtered.

In this study, mashing processes were performed as described in Table 1.

The variation in independent variables directly influenced key wort quality indicators, crucial in determining high-quality wort: extract content, viscosity, and FAN content of wort.

The aim of mashing is to yield as much extract content from the grain as possible, which is further related to the soluble fermentable sugars in wort and is correlated with high concentrations of ethanol produced during fermentation. Wort extract content, which refers to the compounds from finely milled malt that are brought into solution during a mashing process, was determined by MEBAK method 3.1.4.2.2 [25] using Pyknometers on the basis of the official Plato tables.

In this study, high wort viscosity was observed, which is a common occurrence when adjuncts are utilized. Therefore, the commercial enzyme Shearzyme^®^ 500 L was added to the mashes. At the beginning of experiments, 50 µL of enzyme per beaker was used, according to the producer’s recommendations. However, initial enzyme concentration significantly reduced wort viscosity (discussed below). Therefore, in further experiments, enzyme concentration was optimized, e.g., reduced to achieve an adequate wort viscosity as well as to bring down total production costs. The final enzyme concentration was 5 µL per beaker. The viscosity of the wort was measured using a Höppler falling ball viscometer (Brookfield Engineering Laboratories, Inc., Stoughton, MA, USA). Following the MEBAK method 3.1.4.4.1 [25], the amount of time required for a special ball to fall while sinking through a glass tube filled with a wort, between two marks, was measured.

FAN content has been regarded as a superior parameter that represents nitrogen compounds which may be assimilated or metabolized by yeast during fermentation. The problem arises when the most commonly used adjuncts usually decrease the FAN content of wort [27,28]. However, triticale stood out as a cereal that contributes a considerable amount of proteolytic enzyme activity, even in its native form [29]. FAN content in the wort, as reported in MEBAK method 3.1.4.5.5.1 [25], was measured by spectrophotometry. Sample was diluted 1:100. A total of 2 mL of sample was put in a test tube with 1 mL of color reagent and placed in a boiling water bath for exactly 16 min and then cooled to 20 °C for 20 min. Then, 5 mL of dilution solution was added to the test tube, mixed, and absorbance was measured at 570 nm against a reagent blank.

#### 2.2.2. Mashing Process Modelling

In this study, an ANN is proposed as a modeling tool to capture the complex relationships between the input (independent) variables and output (dependent) variables in the mashing process for triticale wort production (Figure 1). For that purpose, Deep Learning Toolbox from Matlab was used.

ANNs are computational models inspired by the structure and functioning of the human brain. They consist of interconnected nodes, or neurons, organized in layers. ANNs learn from data by adjusting the weights of connections between neurons through a training process, enabling them to recognize patterns. The network utilizes mathematical functions, called activation functions, to compute the output of each neuron based on the weighted sum of its inputs.

In this research, a three-layer feed-forward ANN with sigmoid activation function in hidden neurons and linear activation function in output neurons was developed for predicting the characteristics of wort. Network inputs were carefully designed as four indicators with the greatest impact on prediction performance—triticale ratio, enzyme ratio, mashing regime index, and triticale variety index.

The grist included different triticale ratios: 10%, 30%, 50%, and 70%. The enzyme ratio, which denotes the percent value of the concentration of the commercial enzyme Shearzyme^®^ 500 L, was scaled to 50 µL (100%). Based on that, 10 µL quantity was scaled as 20% and 5 µL quantity as 10%. Maintaining uniformity in the range of input data is crucial for better convergence and improved performance. The mashing regime index reflected two different mashing regimes (Congress–1 and Modified–2). The triticale variety index represented two triticale varieties in different forms utilized in the process (unmalted NS Paun–1, unmalted Odisej–2, malted NS Paun–3, and malted Odisej–4). Network outputs were carefully designed as three indicators for wort quality estimation—wort extract content, wort viscosity, and FAN content.

**Figure 2:**
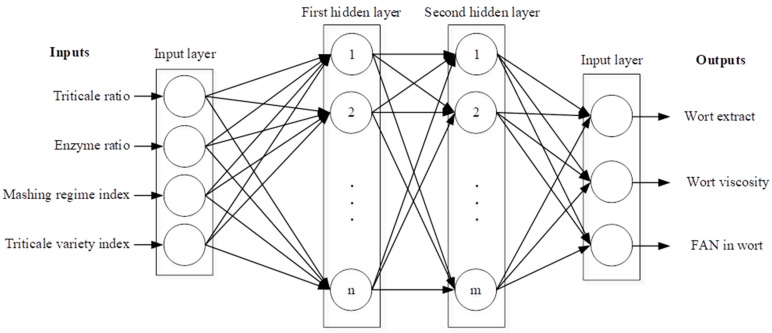
The architecture of the ANN model.

The number of neurons in the first hidden layer is denoted as n and the number of neurons in the second hidden layer is denoted as m. The optimal number of neurons in each layer m and n was determined through systematic experimentation. The selected configuration, obtained through iterative optimization on a validation set, strikes a balance between model complexity and generalization, ensuring optimal performance. The architecture of the model is illustrated in Figure 2.

In this study, three different training functions, namely Levenberg–Marquardt, Bayesian regularization, and scaled conjugate gradient algorithm (implemented in Deep Learning Toolbox from Matlab), were employed to optimize the ANN model using the comprehensive and well-structured dataset.

The Levenberg–Marquardt training function is a widely used method in ANN training. It combines the advantages of both the gradient descent and Gauss–Newton methods, offering robustness and fast convergence. By adjusting the damping factor during the training process, the algorithm effectively balances the stability and speed of convergence, ensuring accurate predictions for the mashing process.

Another training function utilized in this study was Bayesian regularization. This method incorporates Bayesian principles to improve the generalization ability of ANN models. It employs prior knowledge to guide the learning process and prevent overfitting, where the model becomes overly specialized to the training data and fails to generalize to new instances.

The third training function employed in this study was the scaled conjugate gradient algorithm. It is a variant of the conjugate gradient method that adapts the learning rate dynamically during the training process. As a result, it optimizes ANN models by efficiently traversing the error surface, leading to faster convergence and improved prediction accuracy.

By employing three different training functions, the study aimed to thoroughly explore the optimization landscape and identify the most effective approach for training the ANN model. By comparing and evaluating the performance of the ANN models trained with these training functions, valuable insights can be gained regarding the most suitable approach for accurately modeling and predicting the mashing process in triticale wort production.

#### 2.2.3. Mashing Process Model Optimization

In this study, the GA optimization method was proposed as a powerful approach to optimize the input variables in the mashing process for triticale wort production (Figure 1). For that purpose, Global Optimization Toolbox from Matlab was used.

A genetic algorithm is a search heuristic inspired by the process of natural selection. It starts with a population of potential solutions represented as individuals in a population. These individuals undergo evolution through processes such as selection, crossover (recombination), and mutation. The fittest individuals, as determined by a fitness function, are more likely to be selected and pass their genetic information to the next generation. Over successive generations, the algorithm converges towards optimal or near-optimal solutions, mimicking the principles of natural evolution to find solutions to optimization and search problems.

The GA efficiently explored the solution space and converged towards a set of solutions that optimized the desired wort quality objectives. The integration of the GA with the mashing process model, which incorporated ANNs, enabled the optimization of input variables based on their impact on wort quality, offering improved control and efficiency in triticale wort production.

To optimize the mashing process represented by the ANN model, a GA was employed to find the combination of input parameters that yielded the most suitable outputs satisfying the multi-objective function. Through the iterative process of GA optimization, the ANN model was able to converge on the most favorable combination of inputs, resulting in improved predictions of wort production characteristics.

In the optimization process, the input variables triticale ratio, enzyme ratio, mashing regime index, and triticale variety index were constrained only to their natural bounds (between minimum and maximum possible values).

To accurately reflect the importance of the model outputs in the optimization, we introduced penalties that scaled the output of the objective function. By incorporating these penalties, we ensured a balanced consideration of both the primary objective and the influence of the model outputs. Through this approach, our optimization procedure effectively captured the intricate relationships between the inputs, outputs, and penalties, allowing for an informed decision-making process that optimized the primary objective while appropriately accounting for the impact of the model outputs.

The penalties modified the fitness value of each chromosome in the GA optimization process. A higher penalty value indicated poorer fitness, discouraging the selection of chromosomes that violated constraints or deviated from preferred input variable combinations.

As there is no specifically defined range of wort extract content in Brewer’s analytics, in this study, we aimed for wort extract to be in the range of the control sample—100% barley malt (8.57% *w*/*w*) and the highest achieved value in triticale-based wort (8.89% *w*/*w*—70% ratio of variety NS Paun malt without enzyme addition).

The desired value for wort extract was w_e_* is 8.65% *w*/*w* (randomly chosen from obtained wort extract content results which complied with above mentioned criterion), so the penalty function P_e_ for wort extract is as follows:

(1) $$P_{e}=\left\{\begin{array}{cc} w_{e}>8.89 & 10\\ 8.57\leq w_{e}\leq 8.89 & 1\\ w_{e}<8.57,\; & 10 \end{array}\right.$$

where w_e_ is the current real value of wort extract.

The recommended value for wort viscosity w_v_ ranges from 1.5 m·Pas to 1.6 m·Pas [25] and the desired value w_v_* is 1.580 m·Pas (reference value obtained in 100% barley malt wort), so the penalty function P_v_ is as follows:

(2) $$P_{v}=\left\{\begin{array}{cc} w_{v}>1.5 & 10\\ 1.5\leq w_{v}\leq 1.6 & 1\\ w_{v}<1.6,\; & 10 \end{array}\right.$$

where w_v_ is the current real value of wort viscosity.

The recommended value for FAN content in wort w_FAN_ ranges from 110 mg/L to 180 mg/L [25] and the desired value w_FAN_* is 147.99 mg/L (reference value obtained in 100% barley malt wort), so the penalty function P_FAN_ is as follows:

(3) $$P_{\mathrm{FAN}}=\left\{\begin{array}{cc} w_{\mathrm{FAN}}>110 & 10\\ 110\leq w_{\mathrm{FAN}}\leq 180 & 1\\ w_{\mathrm{FAN}}<180,\; & 10 \end{array}\right.$$

where w_FAN_ is the current real value of FAN content.

We formulated a multi-objective optimization problem incorporating three primary objectives. The initial objective was to align the wort extract content with its desired values as accurately as possible. This objective takes into account a penalty P_e_ and can be expressed as follows:

(4) $$F_{e}=P_{e}\left|w_{e}-w_{e}^{*}\right|$$

The second objective was to closely align the wort viscosity with its desired values. This objective takes into account a penalty P_v_ and can be expressed as follows:

(5) $$F_{v}=P_{v}\left|w_{v}-w_{v}^{*}\right|$$

The third objective was to achieve a close match of FAN content with its desired values. This objective takes into account a penalty P_FAN_ and can be expressed as follows:

(6) $$F_{\mathrm{FAN}}=P_{\mathrm{FAN}}\left|w_{\mathrm{FAN}}-w_{\mathrm{FAN}}^{*}\right|$$

By minimizing these metrics during the optimization process, we strived to align the model outputs as closely as possible with their desired values.

By formulating an objective function with penalties, the GA efficiently searches for solutions that optimize the desired wort quality objectives while satisfying the imposed constraints and preferences. This approach provides a comprehensive optimization framework that balances multiple objectives and ensures the feasibility and desirability of the optimized input variable combinations for triticale wort production.

## 3. Results and Discussion

### 3.1. Influence of Triticale Characteristics on Wort Quality

Analysis of the wort was conducted in triplicate, encompassing a total of 384 samples. For the purpose discussing wort quality, the results of all produced triticale wort were compared to the control sample—100% barley malt wort (wort extract content—8.57% *w*/*w*; wort viscosity—1.580 mPa·s; FAN content—147.99 mg/L).

The wort results obtained from triticale mashing in the range of 10–70% in grist, applying both mashing regimes, are shown in Table 2 and Table 3.

At a 10% ratio (as outlined in Table 2), the unmalted triticale sample of the NS Paun variety demonstrated the highest extract value of 8.49% *w*/*w*. Additionally, samples derived from a mixture of triticale malt and barley malt exhibited comparable or even higher extract content when compared to the control sample of barley malt (up to 8.89% *w*/*w* for 70% triticale malt of the NS Paun variety without enzyme addition—Table 2). These findings are in accordance with the results obtained by Cioch-Skoneczny et al. (2019) [12] where a 50% ratio of triticale malt yielded an extract content higher than that obtained in 100% barley malt wort (8.79 and 8.57% *w*/*w*, respectively). The Congress mashing regime, compared to the modified one, showed higher extract content values in all examined samples. Certainly, using triticale in both forms notably influenced the concentration of the wort extract.

At the beginning of the experiments, when 50 µL of enzyme was used, viscosity in all produced wort was significantly reduced, and was lower than the prescribed values (1.5–1.6 m·Pas) [25]. Therefore, in further experiments, enzyme concentration was refined. The final amount was 5 µL, where desirable viscosity was obtained when unmalted and malted triticale were used in 10–50% grist. Overall, the Odisej variety obtained higher viscosity values compared to those produced from the NS Paun variety. It is important to emphasize that compared to the 100% barley malt, all examined samples, in both mashing regimes, had higher viscosity values if the commercial enzyme was not applied. Without the addition of Shearzyme^®^ 500 L, the unmalted Odisej variety showed up to 2.22 mPa·s viscosity, while the unmalted NS Paun variety had much lower viscosity values, even when the highest triticale ratio was applied (1.765 mPa·s—Table 2). When employing unmalted triticale for wort production, the modified mashing process yielded lower viscosity values compared to the Congress mashing method. However, according to MEBAK [25], only the NS Paun variety at 10% ratio in the grist had acceptable values, while none of the wort produced from the Odisej variety fulfilled the above-mentioned range (1.5–1.6 mPa·s). As filtration of the wort is one of the most difficult steps in beer production, controlling the wort viscosity is a crucial moment for a successful brewing process. In this respect, the use of a commercial enzyme was necessary and, as expected, using Shearzyme^®^ 500 L helped decrease viscosity.

Almost all examined malt triticale wort, prepared with the Congress mashing regime, contained FAN contents similar to those of 100% barley malt wort or even higher (Table 2). As expected, FAN content in malt wort, in both mashing regimes, was higher than in wort produced with native triticale, since, during malting, grain components degrade and are more susceptible to enzymes. In this study, the 50% ratio of NS Paun malt showed a similar concentration as the wort produced from 100% barley malt (147.97 and 147.99 mg/L, respectively), which is in correlation with the results obtained by Cioch-Skoneczny (2019) [12].

### 3.2. Dataset Analysis

ANNs operate directly on input–output data, so there must be a sufficient number of data to train the ANN and produce valid results [20]. In this study, a comprehensive dataset consisting of 384 samples was utilized to develop and evaluate the proposed models and optimization methods for the mashing process in triticale wort production. The dataset consisted of carefully collected information on various input (triticale ratio, enzyme ratio, mashing regime index, and triticale variety index) and output (wort quality indicators—extract content, viscosity, and FAN content) variables. Statistical parameters were calculated for all indicators and are given in Table 4.

By utilizing this comprehensive and well-structured dataset, the proposed models and optimization methods could be effectively developed, validated, and evaluated.

The dataset was divided into appropriate subsets for training, validation, and testing. The training subset was used to train the models, allowing them to learn the relationships between the input variables and the corresponding output variables. The validation subset was employed to fine-tune the models and assess their generalization ability. Finally, the testing subset served as an independent evaluation set to objectively measure the performance of the trained models and optimization methods.

The dataset served as a valuable resource for capturing the complex relationships between the input variables and the output variables, facilitating the modeling and optimization of the mashing process for triticale wort production.

The careful curation and division of the dataset into subsets for training (340 samples), validation (36 samples), and testing (8 samples) ensured the reliability and accuracy of the proposed models and optimization methods. By leveraging this comprehensive dataset, the study aimed to advance our understanding of the mashing process and enhance the quality and efficiency of wort production in the brewing industry.

### 3.3. ANN Training Results

The training of ANN models is a critical step in developing reliable and accurate predictions for the mashing process in triticale wort production. The training process involved adjusting the weights and biases of the neurons iteratively using optimization algorithms to minimize the error between the predicted outputs and the actual values in the training dataset.

To evaluate the performance and generalization ability of the trained ANN model, a separate validation dataset was used. The validation dataset consisted of samples that were not included in the training dataset. The model’s predictions for the validation dataset were compared to the actual values, and various metrics, such as mean squared error (MSE) or correlation coefficients, were computed to assess the model’s accuracy and reliability.

A total of 231 different values of neurons in both hidden layers (n and m) were tested. In experiments, the number of neurons ranged in steps of five from ten to sixty neurons in the first hidden layer and from zero to thirty in the second layer (zero represents omitting second layer). ANN had four inputs, so it is not recommended to use a number of neurons in the hidden layer smaller than the number of inputs. Also, a very large number of neurons often leads to network overfitting and can be time-consuming during the training process. The training was repeated ten times for each combination of parameters (three training functions and 231 different numbers of neurons in hidden layers). This yielded 6.930 (3 × 231 × 10) treatments for the ANN. The best training function, the most appropriate number of neurons in the hidden layers, and the best combination of both were determined in these experiments.

**Figure 3:**
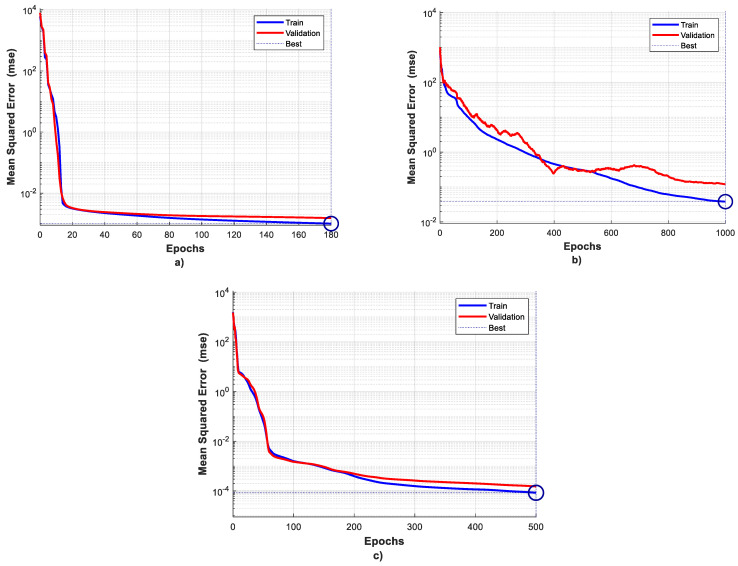
Training performances for the best ANN models obtained for three training algorithms separately; (**a**)—Levenberg–Marquardt training algorithm; (**b**)—scaled conjugate gradient training algorithm and (**c**)—Bayesian regularization training algorithm.

The Levenberg–Marquardt training algorithm yielded optimal results for the ANN model, achieving peak performance with 45 neurons in the first hidden layer and 25 neurons in the second hidden layer. The training concluded after 180 epochs, with a final mean squared error (MSE) of 0.001 on the entire training dataset in the last epoch, as illustrated in Figure 3a.

Similarly, the scaled conjugate gradient training algorithm demonstrated its effectiveness with the ANN model featuring forty neurons in the first hidden layer and five neurons in the second hidden layer. The training process spanned 1000 epochs, and in the last epoch, the MSE on the complete training dataset was 0.04. The convergence pattern is depicted in Figure 3b.

For the Bayesian regularization training algorithm, optimal results were achieved with an ANN model comprising 30 neurons in the first hidden layer and 10 neurons in the second hidden layer. Training concluded after 500 epochs, and in the last epoch, the MSE on the entire training dataset was an impressively low 0.000084. The convergent graph for this algorithm is displayed in Figure 3c.

It is important to highlight that the Bayesian regularization algorithm exhibited superior performance when compared to the other two algorithms, ultimately yielding the most favorable outcomes.

### 3.4. ANN Test Results

To assess the performance and reliability of the trained ANN model, the testing phase was conducted using a set of eight samples. These samples represented real-world scenarios and were carefully selected to cover a diverse range of input variable combinations for the mashing process.

**Figure 4:**
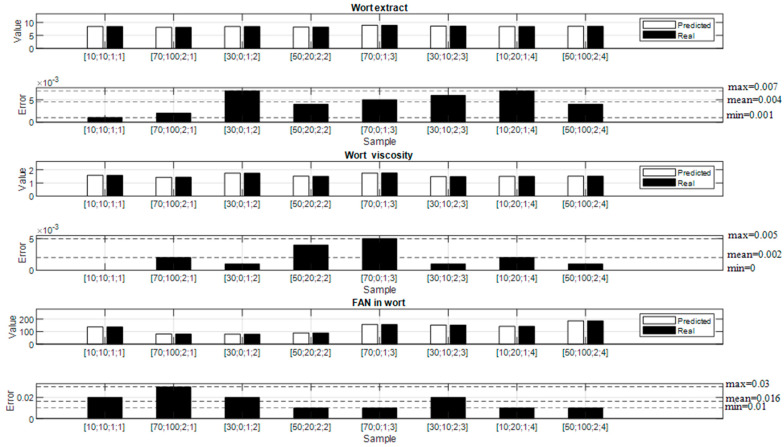
Test results for the proposed neural network model.

During the testing phase, the eight samples were fed into the trained ANN model, and the predicted output variables were compared with their respective actual values. The performance of the model was evaluated by calculating various metrics, such as its mean and minimum and maximum values. The obtained results can be seen in Figure 4.

In Table 5, key metrics for the test results are presented, including minimum and maximum absolute errors, mean absolute error, mean square error, root mean square error, and mean absolute percentage error. These metrics offer a concise assessment of our model’s accuracy, precision, and overall predictive performance on the test dataset.

The results obtained from the testing phase revealed a high degree of accuracy and precision in the predictions of the trained ANN model for all eight samples. The calculated error metrics indicated very small deviations between the predicted and actual values, emphasizing the model’s ability to effectively capture the complex relationships within the mashing process and make accurate estimations of the desired wort quality indicators.

These findings demonstrated the robustness and generalizability of the trained ANN model. The model’s consistent and accurate predictions across the eight samples suggest its reliability in providing precise estimations of the output variables for various input combinations. This indicates the potential of the trained ANN model to be applied in practical brewing scenarios to optimize the mashing process and enhance triticale wort production.

### 3.5. Optimization Results Applying GA

The genetic algorithm was employed to optimize the mashing process for triticale wort production, considering three conflicting objectives simultaneously. The objective functions aimed to address different aspects of the optimization problem, leading to a multi-objective optimization task. The chosen approach for assessing the best set of solutions involved evaluating the Pareto front, which represents the trade-off solutions between the conflicting objectives.

Specific parameters for the GA were configured to address the multi-objective nature of the optimization problem. The population size ranged from 40 to 80 individuals in steps of five and with a crossover rate of 0.8 and a mutation rate of 0.1. Tournament selection was utilized as the selection mechanism, selecting parents based on their fitness with respect to the multiple conflicting objectives.

The GA optimization process involved exploring the Pareto front to identify a set of solutions that provide a balanced trade-off among the conflicting objectives. The termination criteria were based on predefined conditions associated with the convergence of the Pareto front.

The optimization experiment was repeated five times for each combination of parameters, resulting in 40 (8 × 5) optimization attempts. Among the solutions on the Pareto front, one notable set of solutions was found with objective functions values of 0.0949, 0.0131, and 1.6812 for the three conflicting objectives, respectively. These values represent a trade-off that optimally balances the different aspects of the optimization problem.

The corresponding input values for this solution on the Pareto front are shown in Table 6. This solution, achieved with a population size of 50 after 70 generations, is recognized as the best solution among the optimization experiments, demonstrating the most favorable trade-off among the conflicting objectives.

The analysis of the Pareto front provided insights into the solutions that achieved a balance among the conflicting objectives. The selected set of solutions demonstrated a substantial improvement in the overall objective values compared to the initial population, indicating the successful convergence of the GA towards solutions on the Pareto front.

**Figure 5:**
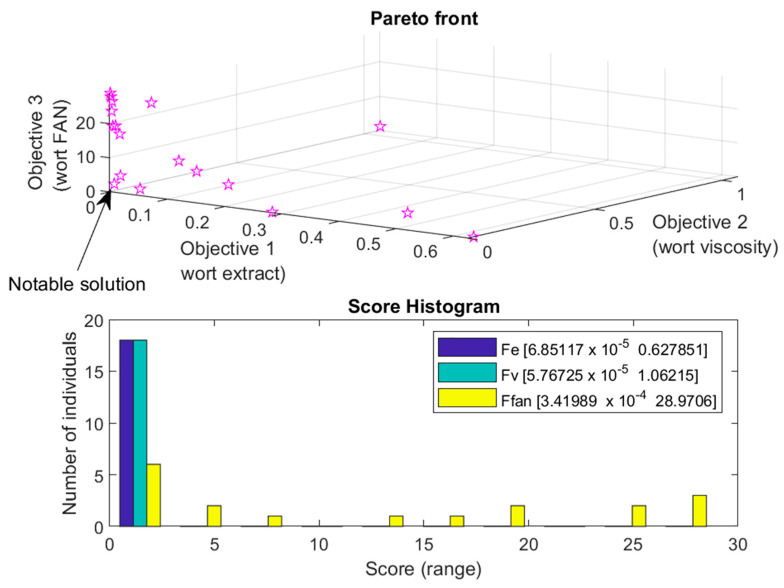
Pareto front for best optimization attempt. Asterisks denote solutions on the Pareto front.

As observed in Figure 5, the upper segment depicts the Pareto front, illustrating the trade-offs and optimal compromises between conflicting objectives. Meanwhile, the lower portion represents a score histogram in Figure 5, offering insights into the distribution of fitness values throughout the optimization process. Together, these visualizations provide a comprehensive understanding of the diverse solutions and the quantitative performance across specific fitness ranges.

The successful convergence of the GA towards optimal solutions is evident from both the Pareto front analysis and the score histogram. The results indicate a substantial improvement in the objective function compared to the initial population, underscoring the efficacy of the multi-objective optimization approach.

The obtained fitness values and the corresponding input–output values highlight the success of the GA in finding an optimized solution for the mashing process in triticale wort production. These results demonstrate the ability of the GA to identify input variable combinations that lead to improved wort quality, as reflected in the obtained output values.

The resulting outputs corresponding to these optimized input variables were measured as 8.65% *w*/*w* for wort extract content, 1.52 mPa·s for wort viscosity, and 148.32 mg/L for FAN content in wort. These values indicated the predicted levels of these wort quality indicators based on the optimized input variables obtained through the GA optimization process.

### 3.6. Experimental Verification

After obtaining the optimization results for the input variables (Table 6), an experimental verification was performed using actual laboratory mashing to validate the effectiveness of the optimized input variable combinations. Wort analyses were performed again in triplicate and the corresponding results are presented in Table 7.

The experimental verification of the real mashing process served as a crucial step in validating the effectiveness of the optimization results and the ANN model. Results from Table 7 demonstrate a very close agreement between the GA-optimized values and the values obtained from the real mashing process and validate the effectiveness in predicting and achieving the desired wort quality.

## 4. Conclusions

The data presented in this work strongly indicate that triticale shows high potential and it is suitable to serve as a brewing adjunct, as it fulfills the most important requirements for wort production. From the presented parameters (Table 2 and Table 3), it is noticeable that triticale influenced the concentration of the wort extract, especially in the form of malt, where extract content was similar to or even surpassed the control sample of barley malt used in research. Regarding wort viscosity, compared to the 100% barley malt, all examined samples had higher viscosity values if the commercial enzyme was not applied. Among the two varieties examined, NS Paun was identified as more suitable in that aspect. The use of a commercial enzyme was necessary and, as expected, using Shearzyme^®^ 500 L helped decrease viscosity which aids wort filtration. FAN content in malt wort, in both mashing regimes, was higher than in wort produced with native triticale.

The utilization of ANNs in triticale wort production has demonstrated its effectiveness in modeling the mashing process. By leveraging the capabilities of ANNs, it becomes possible to accurately predict wort quality based on various input variables, such as triticale variety, triticale ratios, mashing regimes, and enzyme concentrations. The integration of ANNs with GAs further enhanced the optimization process, allowing for the identification of optimal input combinations that minimize specific objective functions related to wort quality. The application of ANNs in the brewing industry represents a significant advancement, enabling brewers to achieve improved control and efficiency in the production of high-quality triticale wort.

The results obtained from the optimization process using a GA and the subsequent experimental verification on the real mashing process have demonstrated the practical applicability of the proposed approach which confirms the potential to enhance the quality and efficiency of triticale wort production.

## Figures and Tables

**Table 1:** Mashing processes applied in the wort production.

Independent variables	Triticale variety	‘NS Paun’ and ‘Odisej’ both in unmalted and malted forms
	Triticale ratio in the grist (%)	10, 30, 50, and 70
	Enzyme quantity (µL)	50, 10, and 5
	Mashing regime	Congress mashing [25]: Initial temperature of 50 °C was maintained for 40 min. The temperature was then increased to 63 °C and held for 45 min. After the addition of a further 100 mL of distilled water, temperature was raised to 70 °C and held for 30 min. Final temperature of 76 °C was maintained for an additional 10 min.
		Modified mashing [26]: Initial temperature of 45 °C was maintained for 30 min before it was increased up to 70 °C at 1 °C/min. After the addition of a further 100 mL of distilled water, the temperature was maintained at 70 °C for a further 60 min.
Dependent variables	Quality parameters	Wort extract content, wort viscosity, and FAN content

**Table 2:** Results of triticale-based wort in 10–70% ratios in grist obtained from Congress mashing.

			Outputs		
	Variety	Enzyme Addition	Wort Extract Content (% *w*/*w*)	Wort Viscosity (mPa·s)	Wort FAN Content (mg/L)
Unmalted	NS Paun	Without enzyme	8.28–8.49	1.626–1.765	64.1–129.42
		50 µL (100%)	8.13–8.38	1.384–1.498	77.23–148.16
		10 µL (20%)	8.22–8.39	1.403–1.587	70.81–141.89
		5 µL (10%)	8.22–8.46	1.576–1.613	67.23–136.37
	Odisej	Without enzyme	8.27–8.47	1.639–2.220	56.0–120.78
		50 µL (100%)	8.22–8.33	1.386–1.495	74.62–139.99
		10 µL (20%)	8.22–8.43	1.401–1.588	67.33–130.03
		5 µL (10%)	8.22–8.45	1.538–1.629	64.24–124.55
Malted	NS Paun	Without enzyme	8.75–8.89	1.500–1.755	130.95–156.12
		50 µL (100%)	8.45–8.80	1.432–1.582	157.43–191.37
		10 µL (20%)	8.49–8.81	1.461–1.583	142.19–189.04
		5 µL (10%)	8.54–8.83	1.468–1.590	146.98–187.97
	Odisej	Without enzyme	8.69–8.78	1.564–1.833	125.80–142.91
		50 µL (100%)	8.39–8.71	1.440–1.570	143.37–186.35
		10 µL (20%)	8.42–8.74	1.498–1.685	140.50–184.46
		5 µL (10%)	8.46–8.77	1.500–1.783	139.76–183.59

**Table 3:** Results of triticale-based wort in 10–70% ratios in grist obtained from Modified mashing.

			Outputs		
	Variety	Enzyme Addition	Wort Extract Content (% *w*/*w*)	Wort Viscosity (mPa·s)	Wort FAN Content (mg/L)
Unmalted	NS Paun	Without enzyme	8.21–8.41	1.596–1.650	71.28–134.67
		50 µL (100%)	8.12–8.36	1.320–1.432	79.49–151.82
		10 µL (20%)	8.12–8.37	1.408–1.491	72.29–147.16
		5 µL (10%)	8.28–8.38	1.419–1.527	69.70–140.07
	Odisej	Without enzyme	8.23–8.44	1.607–2.060	63.76–132.69
		50 µL (100%)	8.20–8.32	1.339–1.446	76.4–144.33
		10 µL (20%)	8.21–8.40	1.415–1.522	68.94–138.31
		5 µL (10%)	8.20–8.42	1.529–1.618	67.04–135.13
Malted	NS Paun	Without enzyme	8.69–8.79	1.518–1.713	141.27–159.19
		50 µL (100%)	8.42–8.74	1.414–1.502	162.38–198.62
		10 µL (20%)	8.48–8.75	1.433–1.529	154.18–192.72
		5 µL (10%)	8.52–8.77	1.430–1.562	150.30–188.19
	Odisej	Without enzyme	8.65–8.74	1.541–1.790	138.30–149.20
		50 µL (100%)	8.38–8.67	1.422–1.563	155.13–192.13
		10 µL (20%)	8.40–8.70	1.460–1.680	150.09–188.90
		5 µL (10%)	8.41–8.71	1.480–1.720	148.50–184.42

**Table 4:** Statistical parameters of research data.

	Name of Parameter	Min.	Max.	Mean	Std. Dev.
Inputs	Triticale ratio (%)	10	70	40	22.39
	Enzyme ratio (%)	0	100	32.5	39.66
	Mashing regime index	1	2	1.5	0.5
	Triticale variety index	1	4	2.5	1.12
Outputs	Wort extract content (% *w*/*w*)	8.12	8.89	8.47	0.2
	Wort viscosity (mPa·s)	1.320	2.220	1.56	0.13
	FAN content (mg/L)	56.0	198.62	130.48	39.32

**Table 5:** Test results metrics.

	Min. abs. Error	Max. abs. Error	Mean abs. Error	Mean Square Error	Root Mean Square Error	Mean abs. Percent. Error
Wort extract	0.001	0.007	0.004	0.00002	0.005	0.05
Wort viscosity	0	0.005	0.002	0.00001	0.002	0.13
FAN in wort	0.01	0.03	0.016	0.0003	0.018	0.015

**Table 6:** The optimized input variables obtained from the GA.

Inputs	Result
Triticale ratio (%)	23
Enzyme ratio (%)	9
Mashing regime index	Congress–1
Triticale variety index	Malted NS Paun–3

**Table 7:** Optimization results applying GA and results obtained from real mashing process.

	Wort Extract Content (% *w*/*w*)	Wort Viscosity (mPa·s)	FAN Content (mg/L)
GA optimization results	8.65	1.52	148.32
Real mashing process	8.63 ± 0.01	1.51 ± 0.02	148.88 ± 0.02

## Data Availability

Data is contained within the article.

## References

[1] Dabija A., Ciocan M.E., Chetrariu A., Codină G.G. (2021). Maize and sorghum as raw materials for brewing, a review. Appl. Sci..

[2] Materna K., Bernhäuserová V., Hasman J., Hána D. (2022). How microbreweries flooded Europe: Mapping a new phenomenon in the beer industry. J. Maps.

[3] Anderson H.E., Santos I.C., Hildenbrand Z.L., Schug K.A. (2019). A review of the analytical methods used for beer ingredient and finished product analysis and quality control. Anal. Chim. Acta.

[4] Yorke J., Cook D., Ford R. (2021). Brewing with unmalted cereal adjuncts: Sensory and analytical impacts on beer quality. Beverages.

[5] Ozatay S. (2020). Recent Applications of Enzymes in Food Industry. J. Curr. Res. Eng. Sci. Technol..

[6] Steiner E., Auer A., Becker T., Gastl M. (2012). Comparison of beer quality attributes between beers brewed with 100% barley malt and 100% barley raw material. J. Sci. Food Agric..

[7] Gomaa A.M. (2018). Application of Enzymes in Brewing. J. Nutr. Food Sci. Forecast..

[8] Cadenas R., Caballero I., Nimubona D., Blanco C.A. (2021). Brewing with starchy adjuncts: Its influence on the sensory and nutritional properties of beer. Foods.

[9] Rosa R.S., Lannes S.C.d.S. (2022). Impact of the use of unmalted adjuncts on the rheological properties of beer wort. Food Sci. Technol..

[10] Kok Y.J., Ye L., Muller J., Ow D.S.W., Bi X. (2019). Brewing with malted barley or raw barley: What makes the difference in the processes?. Appl. Microbiol. Biotechnol..

[11] Ambriz-Vidal T.N., Mariezcurrena-Berasain M.D., Heredia-Olea E., Pinzon Martinez D.L., Gutierrez-Ibañez A.T. (2019). Potential of Triticale (X Triticosecale Wittmack) Malts for Beer Wort Production. J. Am. Soc. Brew. Chem..

[12] Cioch-Skoneczny M., Zdaniewicz M., Pater A., Skoneczny S. (2019). Impact of triticale malt application on physiochemical composition and profile of volatile compounds in beer. Eur. Food Res. Technol..

[13] Hu S., Dong J., Fan W., Yu J., Yin H., Huang S., Liu J., Huang S., Zhang X. (2014). The influence of proteolytic and cytolytic enzymes on starch degradation during mashing. J. Inst. Brew..

[14] Lalor E., Goode D., Whitehurst R.J., van Oor M. (2009). Brewing with Enzymes. Enzymes in Food Technology.

[15] Demeester A., Laureys D., Baillière J., Huys J., Vermeir P., De Leyn I., Vanderputten D., De Clippeleer J. (2023). Comparison of Congress Mash with Final 65 °C Mash for Wort Production with Unmalted Barley, Tritordeum, and Quinoa, with or without Pregelatinization and/or Enzyme Addition. J. Am. Soc. Brew. Chem..

[16] Min W., Jiang S., Liu L., Rui Y., Jain R. (2019). A Survey on Food Computing. ACM Comput. Surv..

[17] Gonzalez Viejo C., Fuentes S., Torrico D.D., Godbole A., Dunshea F.R. (2019). Chemical characterization of aromas in beer and their effect on consumers liking. Food Chem..

[18] Takahashi M.B., Coelho de Oliveira H., Fernández Núñez E.G., Rocha J.C. (2019). Brewing process optimization by artificial neural network and evolutionary algorithm approach. J. Food Process Eng..

[19] Mane S.U., Rao M.R.N. (2017). Many-Objective Optimization: Problems and Evolutionary Algorithms-A Short Review. Int. J. Appl. Eng. Res..

[20] Imandi S.B., Karanam S.K., Nagumantri R., Srivastava R.K., Sarangi P.K. (2023). Neural networks and genetic algorithm as robust optimization tools for modeling the microbial production of poly-β-hydroxybutyrate (PHB) from Brewers’ spent grain. Biotechnol. Appl. Biochem..

[21] Becker T., Enders T., Delgado A. (2002). Dynamic neural networks as a tool for the online optimization of industrial fermentation. Bioprocess Biosyst. Eng..

[22] Dębska B., Guzowska-Świder B. (2011). Application of artificial neural network in food classification. Anal. Chim. Acta.

[23] Zhang Y., Jia S., Zhang W. (2012). Predicting acetic acid content in the final beer using neural networks and support vector machine. J. Inst. Brew..

[24] Hassen E.B., Asmare A.M. (2019). Predictive performance modeling of Habesha brewery wastewater treatment plant using artificial neural networks. Chem. Int..

[25] Methodensammlung der Mitteleuropäischen Analysenkommission (2011). Raw Materials: Barley, Adjuncts, Malt, Hops and Hop Products.

[26] Glatthar J., Heinisch J., Senn T. (2002). A study on the suitability of unmalted triticale as a brewing adjunct. J. Am. Soc. Brew. Chem..

[27] Black K., Tziboula-Clarke A., White P.J., Iannetta P.P.M., Walker G. (2021). Optimised processing of faba bean (*Vicia faba* L.) kernels as a brewing adjunct. J. Inst. Brew..

[28] Puligundla P., Smogrovicova D., Mok C., Obulam V.S.R. (2020). Recent developments in high gravity beer-brewing. Innov. Food Sci. Emerg. Technol..

[29] Goode D.L., Arendt E.K., Bamforth C.W. (2006). Developments in the supply of adjunct materials for brewing. Brewing.

